# Hydrodynamically Lubricated and Grooved Biomimetic Self-Adapting Surfaces

**DOI:** 10.3390/jfb5020078

**Published:** 2014-06-04

**Authors:** Robert L. Jackson, Jiang Lei

**Affiliations:** 1 Mechanical Engineering Department, Auburn University, 1418 Wiggins Hall, Auburn, AL 36849, USA; 2 SKL SVMS, Xi’an Jiaotong University, 28 Xianning West Road, Xi’an 710049, Shaanxi, China

**Keywords:** hydrodynamic, texture, lubrication, adaptive, tilted step bearing, Rayleigh

## Abstract

In many machines and mechanical components, there is a need for new bearing technologies to reduce friction and wear, and provide precision control of motion when the load is varied. This can be provided by electronically controlled actuators and sensors on the surfaces, but then the system reliability can be an issue. In contrast, biomimetic surfaces can be created that adapt mechanically to variations in load. This work uses numerical methods to research the use of self-adapting surfaces for bearings that are based on the deformable nature of biological materials such as articular cartilage. These surfaces are designed to change their profiles to achieve a desired behavior, without any external control. The surfaces change their profile to control the film height and tilt of the bearing to a near constant value for different loads. If the surfaces are tilted, the grooved self-adapting surfaces will also react with a larger restoring moment than a conventional grooved surface. These surfaces could be beneficial to applications where electrical systems and controls are not feasible.

## 1. Introduction

Friction and wear in machine components greatly affect their reliability and efficiency and the integrated systems which employ them. Power supplied from generators, engines and motors can be lost through friction and wear in the various interacting surfaces of a machine, regardless of its scale. Such interacting surfaces can be found in mechanical components such as bearings, gears, bushings, pistons, and seals. There is a growing need for the design of these types of components at smaller and smaller scales, and with higher precision and control capabilities.

**Figure 1:**
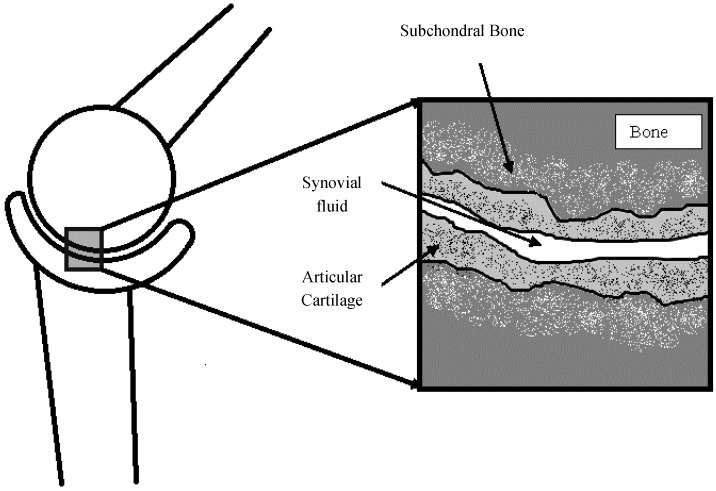
Schematic of a typical synovial joint and the surfaces.

The current work builds on current bearing and sliding surface technologies used to minimize friction and wear between machine component surfaces by researching the use of self-adapting surfaces. This idea stems from the highly efficient and durable tribological surfaces found in biological organisms [1]. In biological organisms, tribological elements such as synovial joints rely on different mechanisms to generate load-carrying capacity than that used in conventional industrial applications. One of the main differences is that in joints there is a soft living layer of articular cartilage between the surfaces that is able to conform and adapt to changes in loading. The biological material in a synovial joint is also very much anisotropic and inhomogeneous, consisting of pores, fibers and other small scale structures, as depicted in Figure 1. The current work uses this concept from nature to create new biomimetic self-adapting surfaces. Lubricated self-adapting surfaces in close contact will adapt their micro-scale profiles to generate hydrodynamic lift and reduce the wear and friction between the surfaces. This will be accomplished by using a system of microstructures that is designed to deform in a way that improves lubrication when changes in load are applied.

Texturing has been proven theoretically and experimentally to be an effective method for improving the tribological performance of lubricated interfacing surfaces. Significant work has been done with micro-scale laser textured surfaces [2,3,4,5,6,7,8,9,10,11]. Those studies have shown that lubricating fluid is pulled into the converging gap formed by the texture when the surfaces slide which generates pressure as the fluid resists compression. However, these textures are usually static or rigid in industrial applications. It is this converging gap that is thought to be primarily responsible for the beneficial results of texturing on all length scales. For instance, at the macro-scale conventional hydrodynamic bearings use a tilted pad or macro-scale geometry, while at the micro and nano-scale, rough surface features can provide lift between machine components like bearings, lip seals, mechanical seals, and pistons [10,11,12,13,14,15,16,17,18,19,20]. The current work will examine how texture performance might change with the controlled deformation of the texture and lubricating film.

**Figure 2:**
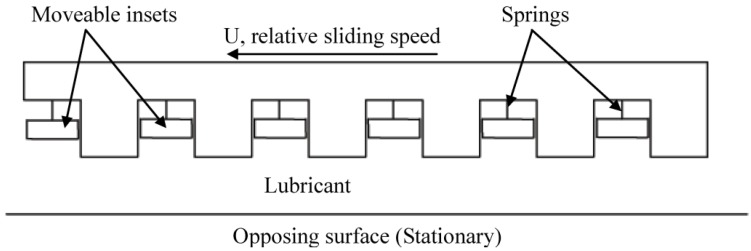
A schematic of a self-adapting grooved surface.

**Figure 3:**
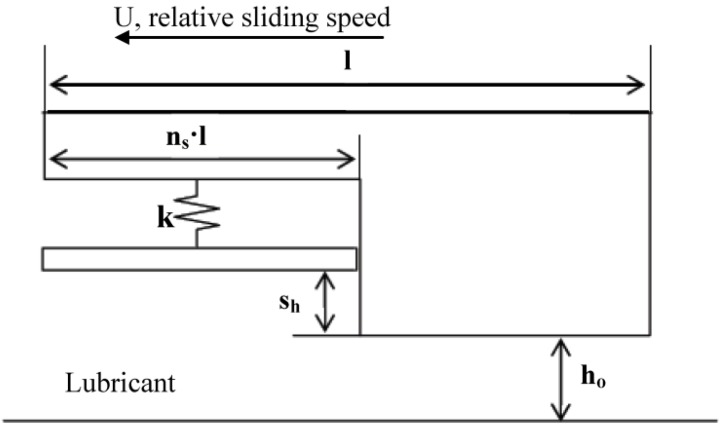
Schematic of the self-adapting step bearing considered in [2].

This investigation of the grooved self-adapting surface (see Figure 2) is based on the previous research of pivoting pad bearings [21] and self-adapting step bearings [22] (see Figure 3). These smart bearings will be designed to change their surface profiles at the nano and micro-scale to achieve a controlled performance to optimize the load support of conventional hydrodynamic bearings (and other lubricated components such as seals and piston rings) for various speeds and film heights.

One of the first works on the self-adapting grooves was on an analytical derivation of the nonlinear spring force and stiffness required for a step bearing to maintain a constant film height [22]. The analytical result is given by

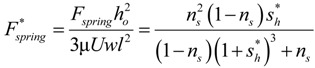
(1)

**Figure 4:**
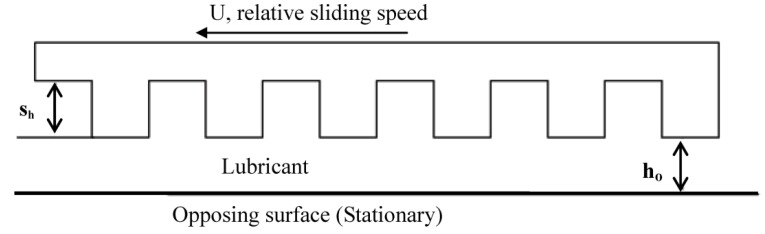
A schematic of a conventional grooved surface.

This force gives the spring force on the inset shown in Figure 2. The spring force is shown plotted *versus* the displacement of the inset, *s_h_* (also the spring deflection) in Figure 4. The force increases with the displacement of the inset, but at a decreasing rate. The required spring force is therefore clearly nonlinear. If this is obtained, a self-adapting step will be created that is able to maintain a nearly constant film thickness for variations in load. If the bearing remains level and the opposing surfaces are parallel, the only variations from this film thickness will be those caused by transient effects until the steady state condition at each load is reached. Additional information can be found in [22]. Note that an alternative spring force that is based on a third order polynomial equation fit to Equation (1) is also provided in [22]. In contrast, the current work also investigates if the surfaces are not in parallel.

Later, Duvvuru *et al.* [23] devised a method to practically apply this concept to a real grooved surface by placing a flexible membrane over the grooves. They used a thin layer or foil to cover micro-scale grooves on a surface. For instance, this could be accomplished by adhering a metal foil onto a surface etched with grooves. In Duvvuru *et al.* [23], a transparent Polydimethylsiloxane (PDMS) surface was fabricated with micro-grooves and covered with a thin, soft layer of PDMS. In that work, the lubrication pressure distribution was solved numerically using a finite volume solution of Reynolds Equation which was also coupled to the deflection of the surfaces via elastic beam deflection theory. Although the stiffness of the springs was not exactly as prescribed by Equation (1), the surface did still maintain a more consistent film thickness than conventional static grooves. Their work showed that there is an ideal thickness and width of the deflecting beams that will result in optimal performance. However, the effects of tilting and moments were not examined in that work.

Most recently, Fesanghary and Khonsari [24] continued this work by examining self-adapting grooves using a mass conserving version of Reynolds Equation and plate deflection theory (*i.e.*, the finite length of the grooves were considered as opposed to assuming them infinitely long in the work by Duvvuru *et al.* [23]). Fesanghary and Khonsari’s work confirmed the previous results but also suggested that the load carrying capacity could be even higher than initially projected. In 2011, Fesanghary and Khonsari [25] also optimized the groove geometry by allowing the width of the grooves to vary along their length.

**Figure 5:**
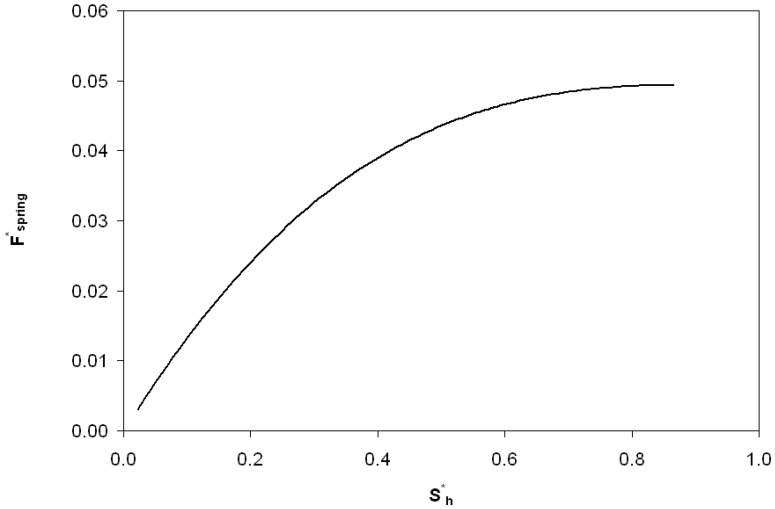
The theoretically predicted spring force required to create a self-adapting step bearing that is able to maintain a constant film thickness for variations in load.

In this work, a numerical and analytical simulation is used to investigate the design of grooved self-adapting surfaces. This results in a bearing that will maintain close to a constant film thickness when under different magnitudes of applied load. Applying the self-adapting grooves will also increase the restoring moment of the surface when it is tilted in comparison to conventional grooved surfaces that do not adapt (see Figure 5). It is very difficult (impossible) to perfectly align two surfaces and this performance quality could be very advantageous to many applications. This aspect of the adaptive surfaces will be one of the main focuses of this work. This has application in machines which require ultra-precision control, such as machining equipment, hard-disk drives, medical devices, and MEMS.

A self-adapting grooved surface bearing may also cost considerably less to manufacture than electronically or hydraulically controlled bearings, but may trade some of their control capabilities for this simplicity and cost effectiveness. In addition, these bearings can be applied to machine elements in which electronics may be difficult to use due to geometrical and kinematical limitations. These bearings will have advantages and disadvantages similar to pivot pad thrust bearing and foil bearings.

## 2. Methodology

**Figure 6:**
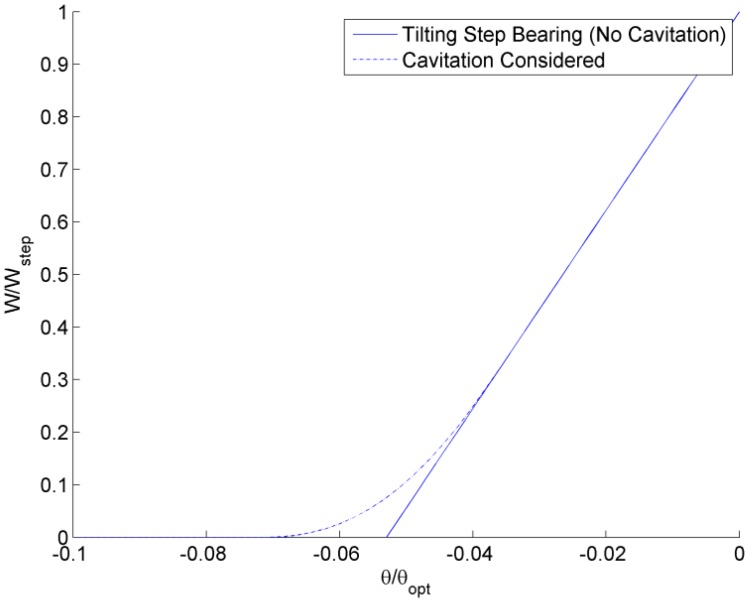
Comparison of two methods to consider cavitation using the numerical solution.

To build upon the previous self-adapting step bearing work [2], the current work first solves analytically the solution for a tilted infinitely long step bearing (see Appendix). This closed form solution then allows for the case of a tilted self-adapting step bearing to be solved (see Figure 6). The closed form solution can then be used to model the case of a tilted grooved surface (where each groove is considered to be an isolated step bearing). This routine can be used to model both tilted conventionally grooved surfaces and the self-adapting grooved surfaces. A numerical model is also constructed for comparison with the analytical model predictions. Therefore, two methods are used to analyze the performance of the grooved surfaces: 1) a closed form isolated groove model; and 2) a full numerical model by solving Reynolds equation.

### 2.1. Normalization Scheme

The following normalization scheme is used to present the results. The loads are normalized by the step bearing solutions [26] (without any tilt):

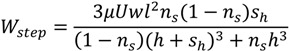
(2)

The moments are normalized by the step bearing solution multiplied by *l*:

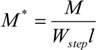
(3)

The angles are normalized by the optimal angle of tilt for a smooth incline bearing [26] which is derived from the optimal step bearing geometry as follows:

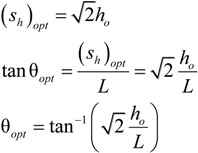
(4)

However, note that *s_h_* is normalized by *h_o_. H_o_* is defined as the desired film thickness and *h_o_* is the minimum film thickness of the bearing. The force and moment calculations are always found for when the minimum film thickness is the desired film thickness (*h_o_ = H_o_*), which is acceptable because the current analysis assumes steady state conditions. In an actual bearing under transient conditions, that minimum film thickness, *h_o_*, will of course vary from *H_o_*, as was considered in [22].

### 2.2. Analytical Model

The solution for a tilted infinitely long step bearing (see Appendix) is used to provide an analytical closed-form model of a tilted self-adapting bearing. This analytical solution is derived from Reynolds equation and is similar to other well-known analytical solutions found in Hamrock [26]. The tilted step bearing solution given in Appendix does not consider cavitation. In the analytical model, cavitation can be considered by using a few different techniques. First, where the analytical model predicts the pressure to be less than zero (atmospheric pressure), it is set to zero. Note that in the current work, all the pressures are solved relative to the atmospheric pressure (*i.e.*, the gauge pressure is solved for), which is a common practice when solving Reynolds equation. This first method is similar to the Half-Sommerfeld Solution and is labeled “Cavitation Considered” in Figure 6. Although cavitation could occur at pressures slightly below atmospheric pressure (negative gauge pressure), the effect is usually considered negligible. Second, a more simple approach is that the overall load support can be set to always be greater than or equal to zero. Predicted loads less than zero are set to zero. This second method does not consider cavitation, until the total load supporting force drops below zero, and therefore does not consider local cavitation. The two methods are compared in Figure 6. It appears that local cavitation is important over only a relatively small range of θ/θ*_opt_* (between approximately −0.07 and −0.04) and so the second method for incorporating cavitation (*W*/*W_step_* ≥ 0) is used in the remainder of the analytical results.

**Figure 7:**
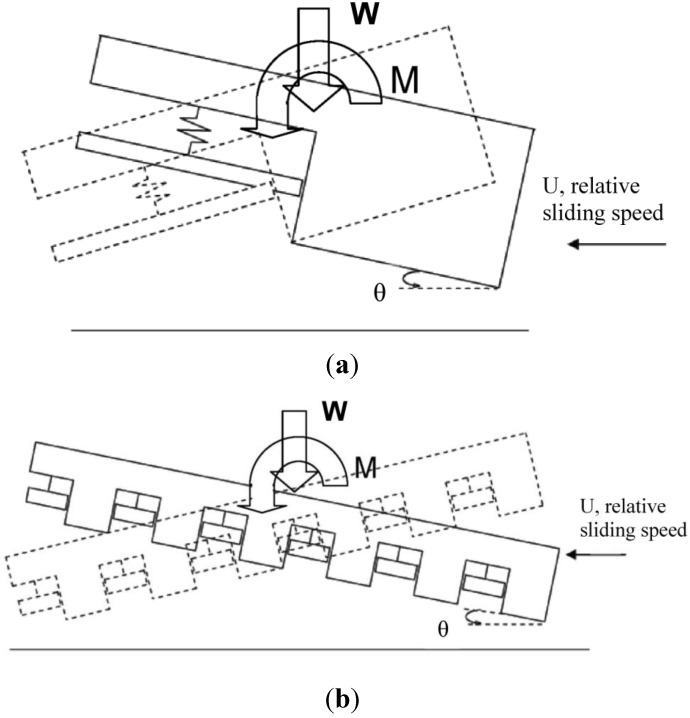
Schematic of (**a**) tilted self-adapting step bearing and (**b**) grooved surface.

Next, the analytical solution for a single tilted step (Figure 7a) is applied to the case of a surface with many grooves (see Figure 7b). It is assumed that the pressure profile of each groove on the surface behaves independently of the neighboring grooves. The numerical model, which is described later, does not make this assumption. This is a good assumption when the diverging portion of the step bearing causes the pressure to reset to zero. However, as will be shown in the numerical results, for a tilted grooved surface, this is not always the case. If the tilt of the overall surface creates a converging gap, then it will tend to form positive pressure which can greatly influence the pressure of each groove.

To apply the single tilted step bearing solution to grooved surfaces, the load generated from each groove is simply summed together for the entire surface:

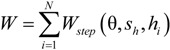
(5)
where *θ* and *s_h_* are given and *h_i_* will vary with an individual grooves location on the surface, such that

*h_i_* = *h*_min_ + *x*θ (6)

where *h_min_* is the local minimum film thickness for one groove segment and *x* is the location on the segment relative to the location of *h_min_*. In the current work, the tilt was applied so that the minimum film thickness did not change, and so was therefore the reference point on the bearing. The results of this methodology, now referred to as the closed form isolated groove model, will be discussed later in comparison to the full numerical model.

### 2.3. Numerical Model

In this paper, the Finite Volume Method is also used to discretize the two dimensional Reynold’s equation. The pressure at the inlet and outlet of the surfaces are set to zero, which is effectively the atmospheric pressure (*i.e.*, we are solving for the gage pressure relative to the atmospheric pressure, which is a common practice in hydrodynamic lubrication calculations [26]). The discontinuity in the surface height in the step is included by satisfying the conservation of flow at that point. Cavitation is considered by limiting all pressures to be zero or positive (*i.e.*, The Reynolds Boundary Condition). More information about the code can be found in [23]. To confirm the validity of the code, the computational result is compared with the analytical solution for the tilted step bearing. The code is then also applied to conventional grooved surfaces and it shows where the analytical solution is not valid. Unfortunately, the numerical model could not be applied to the self-adapting grooved surfaces because it was very difficult for the algorithm to converge (*i.e.*, the steady state deformation of each inset was difficult to reach).

## 3. Results

### Single Groove Results

**Figure 8:**
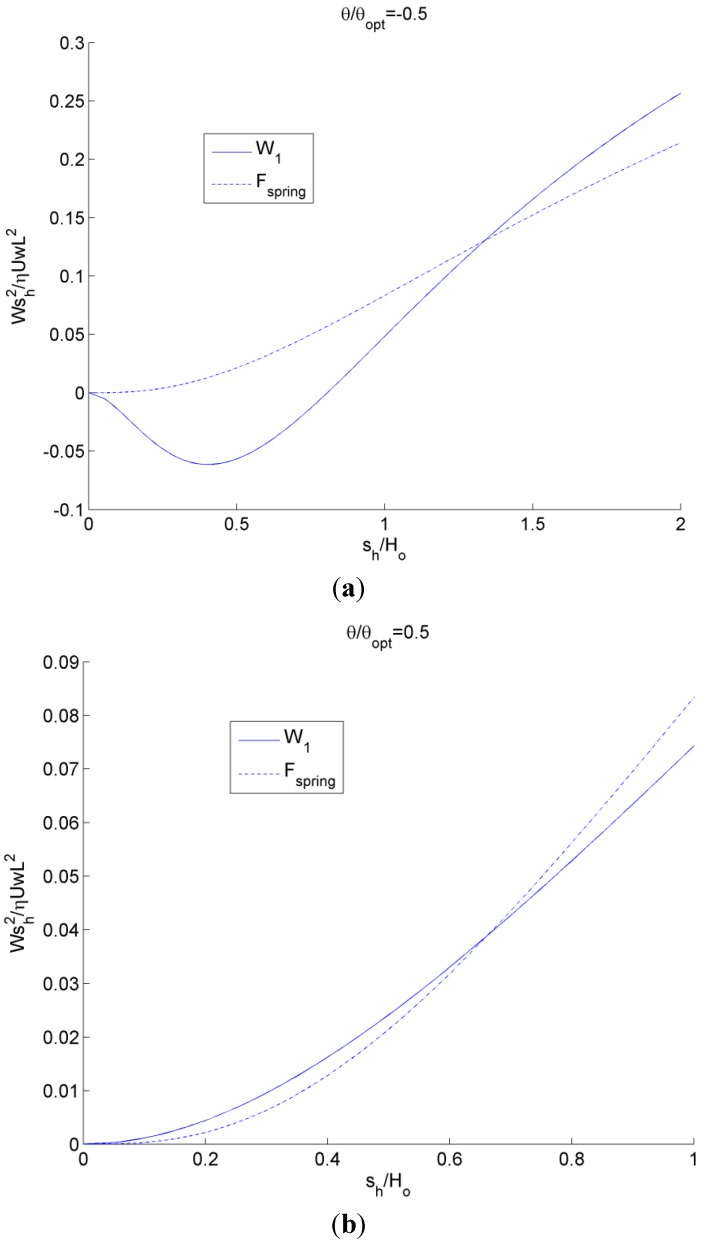
Plot of inset load and nonlinear spring force as a function of inset location using the analytical model for (**a**) negative tilt and (**b**) positive tilt. The steady-state solution is where the lines meet.

Using the nonlinear spring force described by Equation (1) (that provides a prediction for a self-adapting step bearing without tilt with a constant film thickness at steady-state) the effect of tilt is considered (see Figure 8a). These results for the fluid load on the inset, *W*_1_, and the spring force, *F_spring_*, are shown in Figure 8 for a positive tilt of θ/θ*_opt_* = 0.5 and negative tilt of θ/θ*_opt_* = −0.5. The steady-state solution is where the lines meet (*i.e.*, the inset deforms a distance *s_h_* and the bearing geometry is then able to match the fluid lift). For the negative tilt, there is actually a negative fluid lift for small values of *s_h_*, which corresponds to an effective diverging gap in the bearing geometry. Note that no local cavitation is considered here. However, for the positive tilt (*i.e.*, an overall converging gap), the lift never becomes negative and the force of the inset, *W*_1_, and the spring force, *F_spring_*, closely follow each other. In both cases, under steady state conditions (no changes in load or tilt), the inset will converge at a depth at which the two curves meet.

A linear spring can be used instead of the nonlinear spring described by Equation (1). The stiffness of the linear spring is simply set to the stiffness of the nonlinear spring at the desired film thickness. At this desired film thickness, there will be a steady state value of the inset deflection (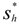)*_i_*. This results in the following equation for the linear spring:

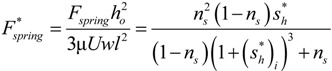
(7)

**Figure 9:**
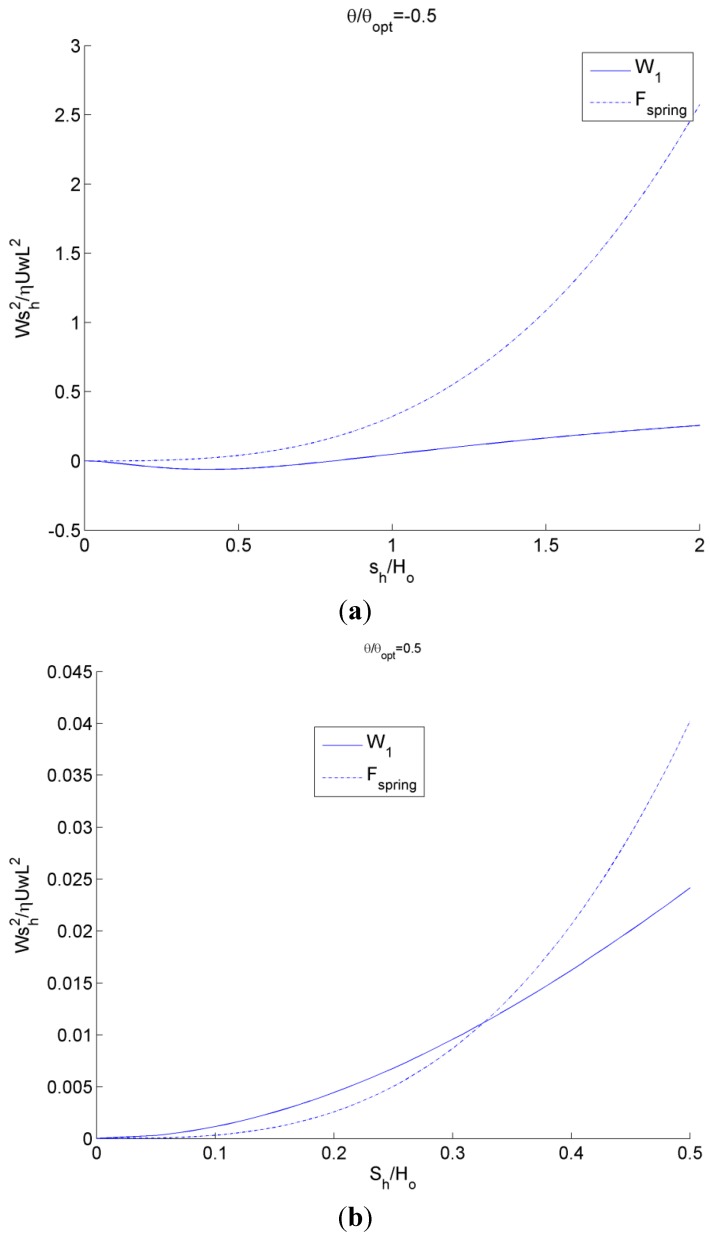
Plot of inset load and linear spring force as a function of inset location using analytical model.

The results when using this linear spring for the fluid load on the inset, *W*_1_, and the spring force, *F_spring_*, are shown in Figure 9 for a positive tilt of θ/θ*_opt_* = 0.5 and a negative tilt of θ/θ*_opt_* = −0.5. Again, the steady-state solution is where the lines meet (*i.e.*, the inset deforms a distance *s_h_* and the bearing geometry is then able to match the fluid load). Unfortunately, the linear spring does not have a solution for negative tilt, except where the inset has no significant deformation. Therefore, it is certainly advantageous to use a nonlinear spring when possible. Recall that a third order polynomial equation fit to Equation (1) is also provided in [22] that provides a performance similar to the nonlinear spring.

**Figure 10:**
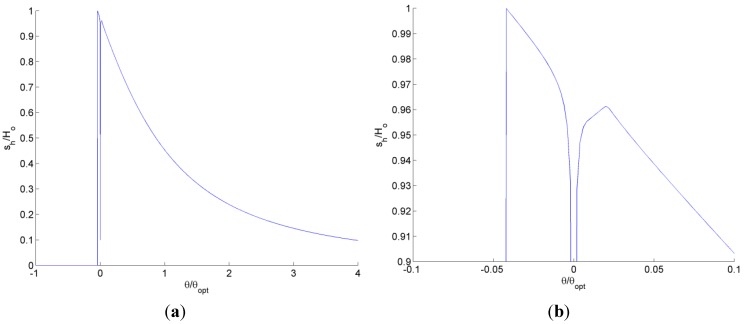
The solved steady-state inset deflection for various tilt angles over (**a**) a large scale and (**b**) a magnified scale.

Solving for the steady state inset deformation for the nonlinear spring case [Equation (1)] for a wide range of tilt angles, we obtain the plot shown in Figure 10. The region of small tilt angles near θ/θ*_opt_* = 0 is enlarged in the figure for clarity. Although the trend is smooth and continuous for most positive tilt angles, as the tilt angle approaches zero, the inset deflection abruptly decreases to the solution for a bearing with no tilt as predicted by Equation (1) and in [22]. Once the tilt angle becomes negative and the bearing possesses an overall diverging gap, the self-adapting inset is only effective until θ/θ*_opt_* ≈ −0.042. However, when the groove is included in an array of grooves over an entire surface, it may actually maintain effectiveness longer, as will be shown later.

**Figure 11:**
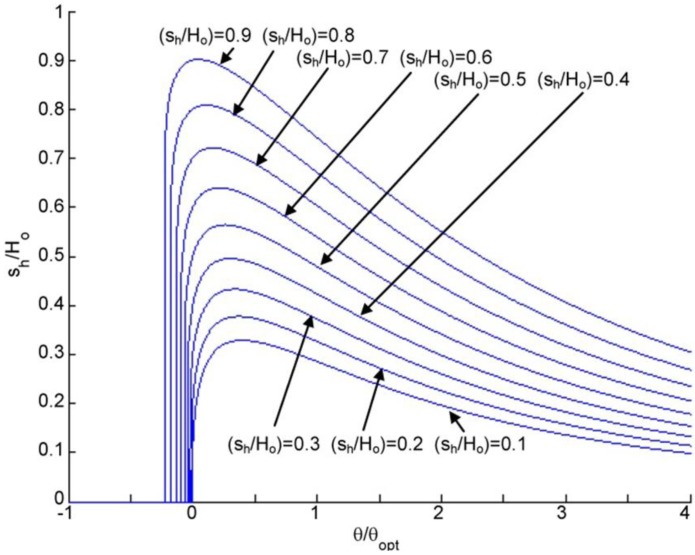
The solved steady-state inset deflection for various tilt angles for a self-adapting step bearing with different linear spring stiffness values.

Likewise, the steady state inset deflection is solved for the single groove using a linear spring as shown in Figure 11. Since the stiffness of the linear spring could be set to match the stiffness of the nonlinear springs at different deflections, the linear spring has been solved for different stiffness’s of the linear spring. Each stiffness is labeled by the equivalent normalized inset deformation of the nonlinear spring. The trend is very similar to the nonlinear spring except that once the inset decreases to nearly θ/θ*_opt_* = 0, it does not recover for any additional amount of negative tilt.

Using the solutions for the tilted self-adapting step bearings with linear and non-linear springs shown in Figure 10 and Figure 11, the overall load carrying performance of the bearing is now analyzed in comparison to static conventional bearings. First, single step bearings will be analyzed and later a surface with a whole array of grooves will be considered.

**Figure 12:**
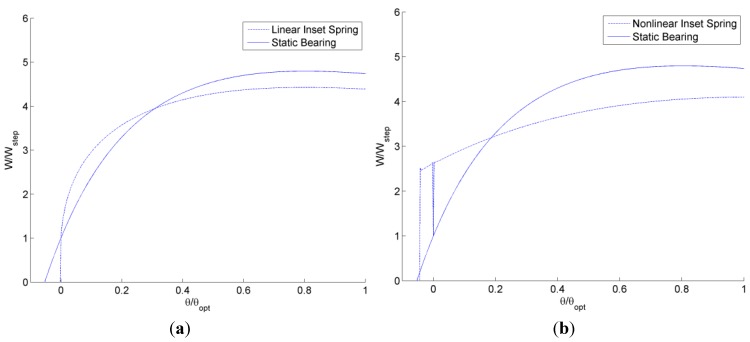
The solved steady-state load carrying capacity for various tilt angles for self-adapting step bearings with a (**a**) linear and (**b**) non-linear spring.

First, the normalized load carrying capacity, *W*/*W_step_*, for a single step designed for (*s_h_*/*h_o_*) = 0.1 is considered using the analytical model described previously (see Figure 12). The geometry of the static conventional bearing is set to the geometry of the self-adapting bearing when no tilt is applied (θ/θ*_opt_* = 0). The predicted load carrying capacity of a self-adapting bearing using a linear spring follows the same general trend of a static bearing, except the static bearing is able to provide load support at some degree of negative tilt angle and also provides higher load support for larger positive tilt angles. However, when a non-linear spring is used, the load carrying performance is improved at small angles (see Figure 12b). The adapting step bearing with the non-linear spring is able to provide significantly higher load support for negative tilts than the other two bearings. Note that the aim of the adapting bearing is not to provide higher load support, but to provide the load support needed to maintain a certain film thickness (with no tilt).

**Figure 13:**
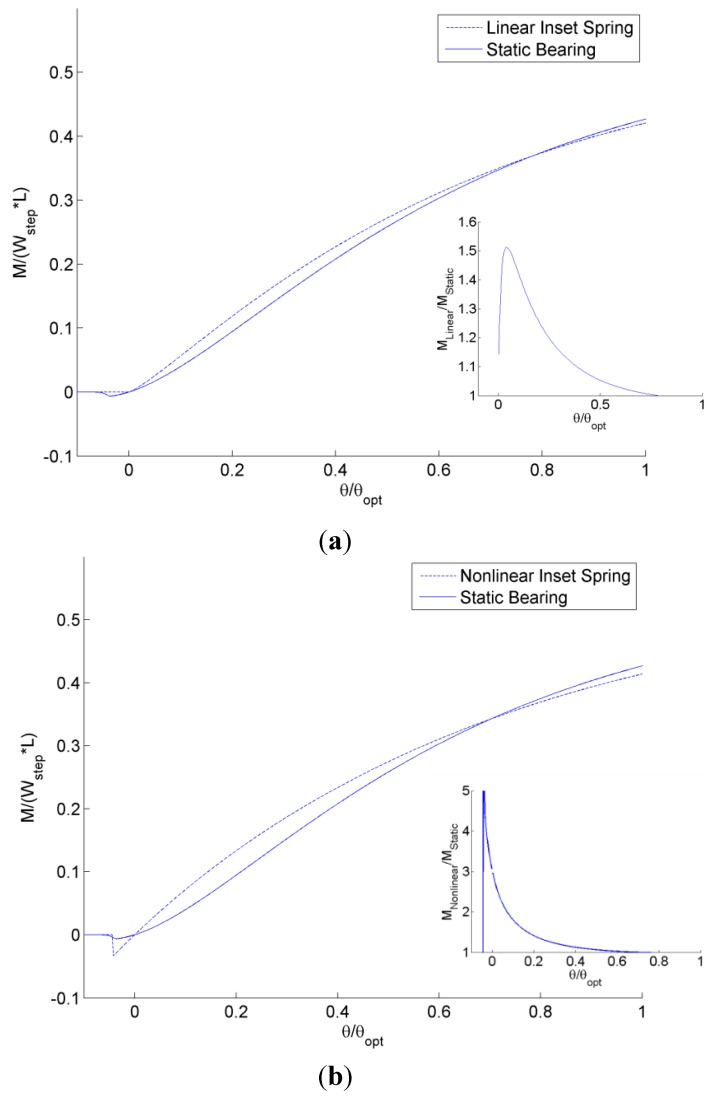
Solved steady-state restoring moments for various tilt angles for self-adapting step bearings with a (**a**) linear and (**b**) non-linear spring.

Next, the predicted restoring moments for a single step designed for (*s_h_*/*h_o_*) = 0.1 is considered using the analytical model described previously (see Figure 13). The restoring moments, *M*, are calculated by numerically integrating the pressure profile of the bearing using the center of the bearing as the fulcrum point. The restoring moments are normalized by the load carrying capacity, *W_step_*, of a conventional step times the length, *L*. A positive restoring moment for θ/θ*_opt_* > 0 is desired to return the bearing to a level status and, likewise, a negative restoring moment is desired for θ/θ*_opt_* < 0. As shown in Figure 13, both bearings provide this general trend except at θ/θ*_opt_* < 0, where all the bearings eventually provide no restoring moment because no pressure is generated and the entire film thickness is undergoing cavitation (*i.e.*, the case of a diverging gap). Both the linear and nonlinear spring self-adapting bearings are better than the conventional static bearing in this regards, but the bearing with the non-linear spring provides the largest restoring moment for θ/θ*_opt_* < 0. The linear inset bearing has a maximum 51% more restoring moment over the static bearing and an angle of θ/θ*_opt_* = 0.0378 (see inset in Figure 13a). The restoring moment of the linear inset bearing does approach the static bearing for smaller angles and larger angles. For the nonlinear spring adapting bearing, an even larger increase is seen. The nonlinear inset bearing has a maximum 652% more restoring moment over the static bearing and an angle of θ/θ*_opt_* = −0.042 (see inset in Figure 13b). As before, the restoring moment of the nonlinear inset bearing does approach the static bearing for smaller angles and larger angles.

## 4. Multiple Groove Results

First, we will analyze the effect of tilt on a static conventional grooved surface with no deformable insets (see Figure 5). The analytical model neglects the effect of tilt angle on the overall pressure distribution over the entire surface (each groove is treated as a separate bearing). A full numerical simulation is therefore used to capture this effect and evaluate how different it is from the analytical model. However, it was difficult for the numerical simulation to include the effects of the adapting insets due to convergence issues. Therefore, we only present the numerical results for a conventional static grooved surface.

Due to cavitation, the grooves of the negatively tilted surface actually behave independently of each other (*i.e.*, the inlet pressure at the start of each groove returns to zero). However, for positive tilt when the overall geometry becomes a converging gap a pressure profile on a larger scale is generated that superimposes on the pressure profiles of the individual grooves and eventually overcomes them as the angle of tilt is increased. The pressure profile appears similar to the solution of an incline bearing for high tilt angles, but the profile is not as smooth due to the grooves.

**Figure 14:**
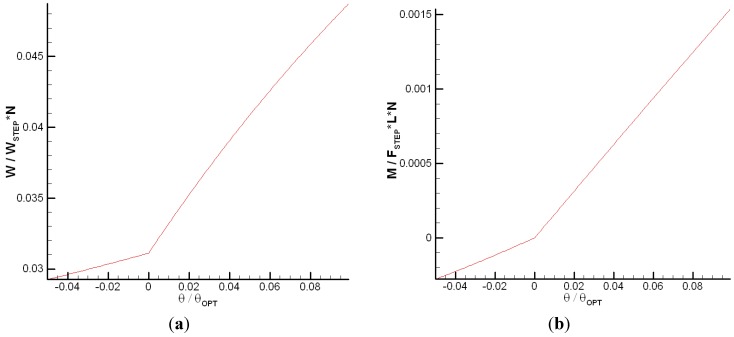
The predicted (**a**) load carrying capacity and (**b**) restoring moment for a tilted conventional static grooved surface.

The numerically predicted pressure profiles are integrated over the surface to find the total load support and also the restoring moment of the static grooved surfaces. These predictions are shown as a function of the tilt angle in Figure 14. The numerical model predictions for negative angles are almost identical to the analytical model (see Figure 13), due to the cavitation isolating each groove. However, the predictions for positive tilt angles are very different (as indicated by discontinuity in slope).

**Figure 15:**
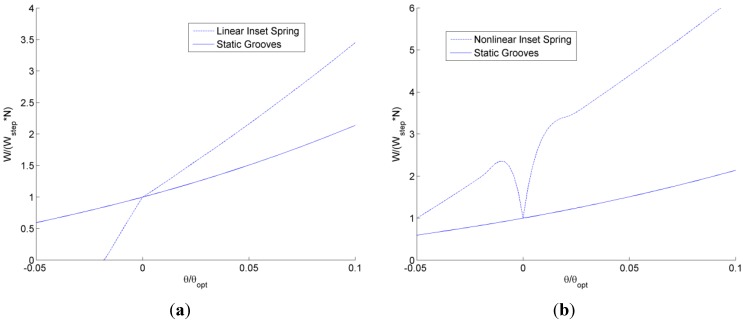
The analytically predicted lift for tilted static and self-adapting grooved surfaces using (**a**) linear and (**b**) non-linear springs.

**Figure 16:**
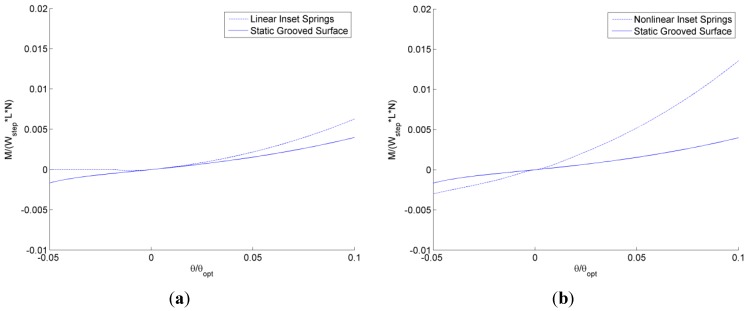
The analytically predicted restoring moments for tilted static and self-adapting grooved surfaces using (**a**) linear and (**b**) non-linear springs.

Next, the self-adapting step bearings are applied as an array of grooves over a surface (see Figure 6) and compared to conventional static grooved bearings using the previously described analytical model for both. The resulting total load, *W*, and the restoring moment, *M*, resulting from integrating the analytically predicted pressure profiles are shown in Figure 15 and Figure 16. In Figure 15, the total load supports for the self-adapting grooved surface using a linear spring and a non-linear spring are shown. Likewise, in Figure 16, the predicted restoring moments are shown for each spring type. One can first see that the trends of the full numerical model predictions (Figure 14) and the analytical model (Figure 15) for the static grooved surface are very similar for both load support and restoring moment.

From Figure 15, it is very clear that the self-adapting grooved surface using non-linear springs performs much better than the one using the linear spring in terms of load support. Actually, the linear spring grooved surface loses load support at negative tilts and will even underperform the static grooved surface. However, the non-linear spring surface will increase the load support for both negative and positive tilts. This could actually help to prevent contact between the surfaces when they are tilted (the minimum film thickness decreases), as this additional load support would increase the overall film thickness between the surfaces until the load support balances with the externally applied load. Similar to the single self-adapting step bearings, there is a sudden decrease in the load support near θ/θ*_opt_* = 0, when the bearing will return to the designed self-adapting behavior for nominally parallel surfaces.

Similarly, the self-adapting grooved surfaces with non-linear springs performed better than both the linear spring adaptive surfaces and the static surfaces (see Figure 16). The grooved surfaces with linear springs actually provided no restoring moment for negative tilts, while the static grooved surfaces did (although for positive tilt the restoring moment of the self-adapting grooved surface was larger), as shown in Figure 16a. The self-adapting grooved surfaces that use the non-linear springs double the restoring moment in comparison to the static grooved surfaces for negative tilts and may triple it or more in the positive tilt range (see Figure 16b). This performance would help ensure that a surface is more parallel to an opposing surface and could be very advantageous in some applications.

## 5. Conclusions

Due to its soft, porous, and inhomogeneous nature, articular cartilage and other biological materials often have an ability to autonomously adapt mechanically to changes in conditions, such as loads, tilts, and misalignment. For industrial applications, these self-adapting qualities can be employed by using surfaces designed to deform in a controlled manner. This results in the concept of self-adapting grooved surfaces for lubricated bearing surfaces. In this work, the concept of a self-adapting step bearing was extended to grooved surfaces with many steps. Analytical and numerical solutions were presented for the cases of a tilted static step bearing, a tilted adapting step bearing, a tilted static grooved surface, and a tilted adapting grooved surface. For all cases, in positive tilt (converging gap) the load carrying capacity increases while in negative tilt (diverging gap), it decreases. Steady-state graphical solutions are provided for the single grooved self-adapting step bearings, which are used to consider the surfaces with many grooves. The analytical model neglects pressure resulting from the converging gap from surface tilt. A restoring moment is present for the grooved surfaces which appears to be enhanced by the adapting grooves. However; the restoring moment is much larger for positive tilts than negative tilts.

## Figures and Tables

**Figure A1:**
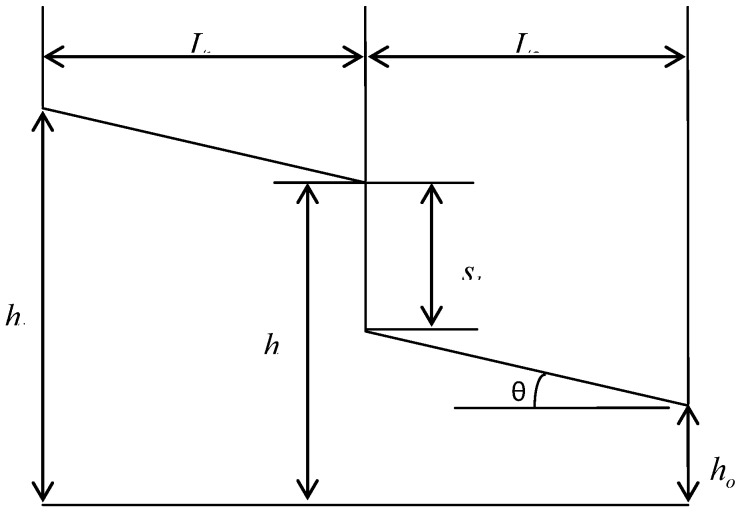
Schematic of a tilted step bearing geometry.

## Nomenclature

*F_spring_*: spring force
normalized spring force
*M*: restoring moment from fluid pressure
*W*: total fluid lift force
*W*_1_: fluid lift force on the inset of a step bearing
*W_step_*: fluid lift force for a single groove on a grooved surface
*H_o_*: desired film thickness
*h*: film thickness
*h_i_*: local film thickness on groove segment *i*
*h_min_*: local minimum film thickness of one groove section
*h_o_*: minimum film thickness
*k*: spring stiffness coefficient
*l*: bearing length or one groove segment length
*L*: grooved surface length
*M*: restoring moment
*M^*^*: normalized restoring moment
*N*: number of grooves on surface
*n_s_*: bearing step length ratio (step length/total length)
*p*: fluid pressure
*s_h_*: bearing step inset height
, *s_h_*: normalized by *h_o_*
*U*: sliding speed
*w*: bearing width
*x*: location on a groove segment relative to the location of *h_min_*
µ: dynamic viscosity
θ: tilt of the bearing surface
θ*_opt_*: optimal tilt of an incline bearing

## Appendix

### A.1. Tilted Static Step Bearing Solution

The slope of the tilted surface could be written as

(A1)

For the first part of the step bearing,

(A2)

which can be used to solve for the pressure by integrating as follows

(A3)

For the second part of the step bearing, similarly,

(A4)

The boundary conditions are given as

1) At the inlet, *h* = *h_i_*, *p*_1_ = 0;
2) At the outlet, *h* = *h_o_*, *p*_2_ = 0;
3) At the step, *h* = *h_s _* or *s_h_*, *p*_1_ = *p*_2_ and *q*_1_ = *q*_2._

Boundary condition 1 results in

(A5)

Boundary condition 2 results in

(A6)

Boundary condition 3 results in

(A7)

Considering mass conservation at the step:

(A8)

and substituting results in

(A9)

Simplifying Equation (A9) results in

*A*_2_ = *A*_1_ + 6*ηS_h_U* (A10)

Substituting Equation (A10) into Equation (A7) results in

Set , and substitute to give

(A11)

Set , and then substituting gives

(A12)

Pressure *p*_1_ and *p*_2_ are then calculated by Equations (A3) and (A4)

The load of the tilted step bearing in each segment are then:

(A13)

Similarly,

(A14)

Then, the total load support provided by the tilted step bearing is: *W* = *W*_1_ + *W*_2_.

It should be noted that the analytical equations above do not consider the effect of cavitation on the tilted step bearing, which could occur for large negative tilts (*i.e.*, a diverging gap).

### A.2. Tilted Self-Adapting Step Bearing Case

As shown by the analytical solution above, when the step bearing is tilted, the pressures and loads will change. For the self-adapting step bearing with a moveable inset, the tilt will also affect the location of the inset and the performance of the bearing. To consider this case, the load produced on a tilted step bearing over the first section of the bearing, *W*_1_, can be equated with the spring force:

(A15)

where the spring constant, *k*, can be either a function of *s_h_* (nonlinear spring) that results in the bearing maintaining a constant film thickness or a constant value (linear spring). These results are shown in Figure 7, Figure 8, Figure 9 and Figure 10.
